# Correction

**Published:** 1870-10

**Authors:** 


Correction.—The case of Ovariotomy reported in the September number of The Examiner, and credited to "M. A. Chase, M.D., of Siruoza, Wis.," should have been credited to H. A. Chase, of Viragua, Wis.

In the record of proceedings of the Illinois State Medical Society, the name of Dr. O. Everett, of Dixon, is printed Dr. O. *Evarts,* and that of Dr. George W. Hewett, of Franklin Grove, is made to read George W. *Heath.* We have read manuscript for many years, but we continue occasionally to misinterpret the names of both men and places.

